# Multiscale modeling and simulation for anomalous and nonergodic dynamics: From statistics to mathematics

**DOI:** 10.1016/j.fmre.2024.12.024

**Published:** 2025-01-11

**Authors:** Heng Wang, Xuhao Li, Lijing Zhao, Weihua Deng

**Affiliations:** a School of Mathematics and Statistics, State Key Laboratory of Natural Product Chemistry, Lanzhou University, Lanzhou 730000, China; b School of Mathematical Sciences, Anhui University, Hefei 230601, China; c School of Mathematics and Statistics, Northwestern Polytechnical University, Xi’an 710129, China

**Keywords:** Multiscale modeling, Anomalous and nonergodic dynamics, Statistical observables, PDE analyses, Scientific computation, Deep learning

## Abstract

In recent decades, anomalous and nonergodic diffusions are topical issues in almost all disciplines. In 2004, the phrase “anomalous is normal” was used in a title of a Physical Review Letters (PRL) paper, which reveals that the diffusion of classical particles on a solid surface has rich anomalous behavior controlled by the friction coefficient, meaning that anomalous diffusion phenomena are ubiquitous in the natural world. This review article first builds the microscopic models (stochastic processes) to describe the experimentally observed phenomena of the motion of the physical/abstract particles under the frameworks of continuous time random walks, Langevin picture, subordinated strong Markov process, etc. Beyond directly conducting statistical analyses on these stochastic processes, we target on delving into these microscopic models to uncover their physical mechanisms and digging out their potential applications. According to the application scenarios and the research requirements, we design the appropriate statistical observables, e.g., the position of the particles, functional of the trajectories of the particles, probability of the first passage time, escape probability, etc. Then, we derive the governing equations of the probability density functions of the statistical observables. We do the mathematical studies on the equations, including well-posedness and regularity analyses, designing numerical schemes, performing numerical analyses, etc. Finally, we present the applications of the models in chemistry and biology, and propose future prospects in this research field.

## 1 Introduction

In nature, “motion” is constantly occurring in a variety of forms. As the process of gene transcription initiates, RNA polymerase moves forward along the DNA synthesizing mRNA and exhibits three different states of motion: transcription extension, backtracking and backtracking recovery [[1], [2], [3]]. Molecular motors operate akin to efficient couriers, tasked with the precise delivery of the newly synthesized mRNA to target sites [4]. The movement of neurotransmitter receptors on the postsynaptic membrane displays an anomalous diffusion of the two states as a consequence of the confinement of nanoclusters [5]. Ligand-receptor interactions have been observed to trigger skipping in three-dimensional media and sliding mechanisms on two-dimensional living cell membranes [6]. Telomeres, special structures at the ends of chromosomes, act as sentinels guarding against genomic instability, yet they are subject to gradual shortening with each cell division [[7], [8]]. In the processes of polymerization and depolymerization, polymers exhibit Brownian non-Gaussian kinetic characteristics [9]. In environments where molecules are densely packed, tracer polymers exhibit anomalous diffusion [10]. The irregular connectivity of pore spaces gives rise to anomalous behavior in fluid flow and chemical transport processes [11]. In the broad scope of ecology, the movement of animals across extensive spatial and temporal scales frequently exhibits anomalous dynamical characteristics [[12], [13]]. To elucidate the essence of these natural phenomena and uncover the underlying physical mechanisms, scientists employ statistical and mathematical tools to quantify the dynamical behaviors. Methods for quantitative modeling across multiple scales are primarily categorized into two types: one focused on simulating dynamics at the microscale, and the other dedicated to deriving or establishing evolutionary equations at the macroscale.

There are two commonly used modeling frameworks, the continuous time random walk (CTRW) model and the Langevin equation. The CTRW model [14] assumes that the process of particle motion is a wait-jump periodic process, and that the waiting time $\tau_{i}$ and the jump length $\xi_{i}$ are two random variables satisfying the joint probability density function (PDF) $\phi \left(x,t\right)=E\left[\delta \left(\xi_{i}-x\right)\delta \left(\tau_{i}-t\right)\right]$. Therefore, the probability densities for the waiting time and the jump length are $\psi \left(t\right)=\int_{-\infty }^{+\infty }\phi \left(x,t\right)dx$ and $\eta \left(x\right)=\int_{0}^{+\infty }\phi \left(x,t\right)dt$, respectively. When the waiting time is independent of the jump length, one can obtain the decoupled form ϕ(x,t)=η(x)ψ(t).

**Fig. 1:**
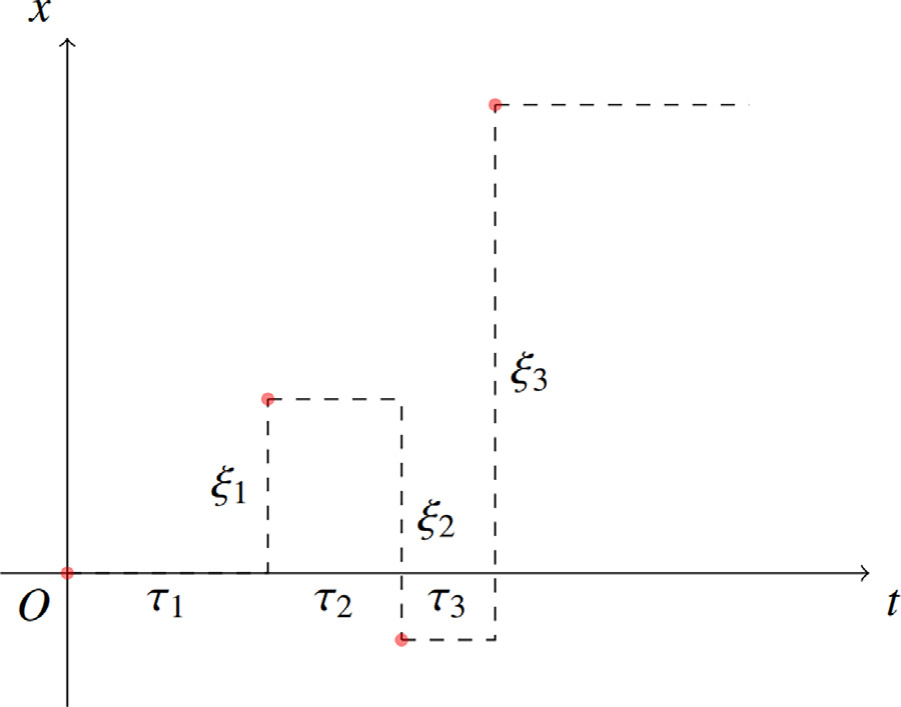
**Particle trajectory of the CTRW model**.

For a given ϕ(x,t), the particle trajectory can be plotted as in Fig. 1. The CTRW model has proven to be a robust and effective tool for quantifying anomalous contaminant transport in porous and faulted geological formations [15]. The CTRW model with a “long memory” waiting time distribution has demonstrated its effectiveness in the financial sector. Assuming that the reaction waiting times for promoter transitions, mRNA synthesis and degradation follow a general distribution, J. Zhang et al. coarsely derive an iterative equation for the moment-generating function of copy number of mRNA, which conveniently calculates mRNA raw and binomial moments of any order [16]. E. Roldán et al. examine the distribution of recovery time by describing the kinetic behaviour of RNA polymerase during the retrospective recovery phase as a CTRW [17]. Subsequently, W. H. Deng et al. expand upon the CTRW model by introducing the multi-internal states modeling approach [[18], [19]] and the alternating states modeling method [[20], [21]], and then also establish the Lévy walk model in non-static media [22]. S. Fedotov et al. establish a two-state nonlinear CTRW model to depict tumor-cell migration and proliferation invasion [23]. An intriguing addition to the models is the stochastic resetting process, which mimics the behavior of reverting to the starting point to initiate a new search after an unsuccessful attempt [[24], [25]].

Langevin’s equation is an application of Newton’s second law “F=ma” to Brownian particles, which has the form [26]

(1) $$m\frac{d^{2}x\left(t\right)}{dt^{2}}=-m\gamma \frac{dx(t)}{dt}+\xi_{1}\left(t\right),$$

where x(t) is the position of the particle at time t, m is the mass of the particle, $-m\gamma \frac{dx(t)}{dt}$ is the frictional force, and $\xi_{1}\left(t\right)$ is the random force (Gaussian white noise, due to the random collisions of the surrounding molecules). When the random force is no longer white, the general form of the Langevin equation can be written as

(2) $$m\frac{d^{2}x\left(t\right)}{dt^{2}}=-m\int_{t_{0}}^{t}\gamma \left(t-t^{\prime }\right)\frac{dx(t^{\prime })}{dt}dt^{\prime }+\xi_{2}\left(t\right),$$

and the fluctuation-dissipation theorem gives the relationship between the friction coefficient and the random force [27]. A. D. Viñales et al. introduce a Mittag-Leffler correlated random force leading to anomalous diffusion [[28], [29]]. T. Sandev et al. study the analytical form of the relaxation functions for the three-parameter generalized Langevin equation [30]. The general form of the Langevin equation, where the friction term is represented by the regularized Prabhakar derivative, is discussed in [31], and the form incorporating a tempered memory kernel is considered in [32]. Analyzing the “social forces” felt by pedestrians allows us to model their dynamic behavior as a set of nonlinear coupled Langevin equations [33]. Reference [34] presents the Langevin equation for polymers engaged in polymerization/depolymerization reactions, by establishing a random diffusion coefficient that correlates with particle size. Reference [35] presents the Langevin equations for continuous time Lévy walks. X. D. Wang et al. present the Langevin description of the Lévy walk [36]. Y. Chen et al. then provide the Langevin description of the Lévy walk with memory [37] and examine the impact of an external force [[38], [39]]. By making a stochastic scale variation of time, one can construct the time-changed (subordinated) Langevin equation [[40], [41]]. The path properties of the subordinated Brownian motion have been investigated in [42]. Additionally, Ref. [43] has considered the time-changed fractional Brownian motion, discussing the moments, correlation structure, and so on. References [[44], [45], [46]] study the Langevin equations for the subordinated Brownian noise of the tempered Mittag-Leffler memory kernel. Let $S_{\alpha }\left(t\right)$ denote the strictly increasing α-stable Lévy process (subordinator) with the well-known formula for its Laplace transform $\langle e^{-kS_{\alpha }\left(t\right)}\rangle =e^{-tk^{\alpha }}$. Define $E_{\alpha }\left(t\right)=\inf\left\{\tau :S_{\alpha }\left(\tau \right)>t\right\}$, which denotes the left inverse of $S_{\alpha }\left(t\right)$. Then there are scale limit relationships between the CTRW models and the subordinated Brownian motions [[40], [47]], as shown in Table 1.

**Table 1:** **The scale limit relationships between the CTRW models and the subordinated Brownian motions**.

CTRW model	subordinated Brownian motion
$\langle \tau_{i}\rangle <\infty ,\;\langle \xi_{i}^{2}\rangle <\infty$	B(t)
$\langle \tau_{i}\rangle <\infty ,\;\xi_{i}∼{\left|x\right|}^{-2\beta -1}$	$B(S_{\beta }\left(t\right))$
$\tau_{i}∼t^{-\alpha -1},\;\langle \xi_{i}^{2}\rangle <\infty$	$B(E_{\alpha }\left(t\right))$
$\tau_{i}∼t^{-\alpha -1},\;\xi_{i}∼{\left|x\right|}^{-2\beta -1}$	$B(S_{\beta }\left(E_{\alpha }\left(t\right)\right))$

Based on the microscopic model, partial differential equations (PDEs) governing the probability distribution of particle positions can be derived by integral transformations [[40], [48], [49], [50]]. It is also possible to derive the PDEs from the macroscopic scale according to physical assumptions. Furthermore, it is also possible to derive equations that describe the probability distributions governing the macroscopic statistical properties of physical processes, which include the position functional of the particle, the probability density of the mean first passage time, and even the escape probability [[34], [51], [52], [53]].

For the PDEs mentioned above, researchers also discuss the well-posedness and regularity of the solution, as well as develop the corresponding computational methods. The traditional numerical computational methods, such as the finite difference method, the convolution integral method, and the variational method [[54], [55], [56], [57], [58], [59]], provide effective and highly accurate approximations for the solutions of PDEs, but their computational complexity grows exponentially with the dimensionality [59]. With the growth of data resources and computational power, deep learning has become an important tool for solving high-dimensional problems in scientific research. In recent years, a large number of deep learning-based PDE solvers have been developed, most of which are inspired by traditional methods. In 2018, W. E et al. develop the deep Ritz method, which uses deep learning to solve variational problems corresponding to PDEs [60]. Least squares-based deep learning methods include the deep Galerkin method [61] and physics-informed neural networks [62], which train models by minimizing the squared residuals of PDEs. Weak adversarial networks [63] offer an approach to tackle the weak formulations of high-dimensional PDEs utilizing adversarial learning techniques. J. Han et al. propose a deep learning method for solving parabolic PDEs based on backward stochastic differential equations (BSDEs), called the deep BSDE method [[64], [65]]. References [[66], [67]] provide posterior estimates of the deep BSDE method. Subsequently, H. Wang et al. develop the deep BSDE method for solving infinite-dimensional coupled polymer diffusion systems [68].

Based on the aforementioned discussions, this paper provides a comprehensive review of the research progress in multiscale modeling of anomalous non-ergodic dynamical systems found in nature and offers insights into future research avenues. The review is structured as follows: In the second section, we delve into microscopic models, derive the PDEs that govern the probability distributions of various statistical observables, and present some theoretical statistical analysis results. The third section is dedicated to the theoretical analysis and computational methods for the macroscopic equations. In the fourth part, we showcase some applications of the developed models in physics, biology, and engineering. Lastly, we propose potential directions for future research prospects.

## 2 Mathematical modeling

This research focuses on the motion of real physical particle or abstract “particle”, for example, the fluctuation of the financial market. So the first step is to use mathematical language to describe the motion, that is, build the microscopic model (stochastic process) to characterize the motion. Three types of microscopic models will be discussed here, i.e., CTRW, Langevin equation, miscellaneous ones, including subordinated Brownian motion, alternating process, and resetting process, etc. Beyond conducting direct statistical analyses on these stochastic processes, we target on delving into these microscopic models to uncover their physical mechanisms, and digging out their potential applications. To accomplish this, we initiate by designing the corresponding statistical observables and deriving the governing equations that dictate the probability distributions of these statistical observables.

### 2.1 Microscopic models

#### 2.1.1 Continuous time random walks

In mathematics, CTRW is a stochastic process with arbitrary given distributions of jump lengths and waiting times, originally discussed by E. W. Montroll and G. H. Weiss [[14], [69]]; and the CTRW is first applied to physical systems by H. Scher and M. Lax [70], where the random variables of waiting times and displacements are independent and identically distributed, respectively. For the past several decades, CTRW model is used to describe different kinds of anomalous systems, ranging from amorphous semiconductors to DNA molecules.

The CTRW model with multiple internal states is built in [19]. We introduce multiple internal states to characterize some natural phenomena, such as, traps in amorphous semiconductors and ionic currents in cell membranes. Each internal state corresponds to a distribution of waiting time and jump length, and the transitions between internal states are described by a Markov chain with a transition matrix M. The dimension of matrix M is n×n, where n is the number of internal states. The element $m_{ij}$ of matrix M represents the transition probability from state i to j.

Lévy walk is a CTRW model with the jump length determined by the generated waiting time, i.e., for the waiting time $\tau_{i}$, the jump length equals to $v\tau_{i}$, in which v is the speed of the particles. The Lévy walk with multiple internal states is built in [18], having the same structure as the CTRW one.

#### 2.1.2 Langevin equations

In physics, “external potential” refers to the potential energy imposed on a physical system by external factors or environment, generally denoted as V(x). The Langevin picture for depicting external potentials offers significant advantages compared to the CTRW model. The dynamical model of a Brownian particle subject to an external potential can be represented in the Langevin equation, that is,

(3) $$m\frac{d^{2}x\left(t\right)}{dt^{2}}=-\nabla V\left(x\left(t\right),t\right)-m\gamma \frac{dx(t)}{dt}+\xi_{1}\left(t\right),$$

where V(x,t) is the external potential and γ is the friction coefficient. If considering the delayed effect of friction, one can use a natural generalization of the Langevin equation with external potential, known as the generalized Langevin equation

(4) $$m\frac{d^{2}x\left(t\right)}{dt^{2}}=-\nabla V\left(x\left(t\right),t\right)-m\int_{0}^{t}\gamma \left(t-t^{\prime }\right)\frac{dx(t^{\prime })}{dt}dt^{\prime }+\xi_{2}\left(t\right).$$

It is important to note that $\xi_{1}\left(t\right)$ and $\xi_{2}\left(t\right)$ in Eqs. 3 and 4 are different. Since both of them are internal noises, according to the fluctuation-dissipation theorem [27] (friction and random driving forces come from the same source), their correlation functions are

$$\delta \left(t\right)=\frac{1}{mk_{B}T}E\left[\xi_{1}\left(t^{\prime }\right)\xi_{1}\left(t^{\prime }+t\right)\right]$$

and (∇V(x,t)≡0)

$$\gamma \left(t\right)=\frac{1}{mk_{B}T}E\left[\xi_{2}\left(t^{\prime }\right)\xi_{2}\left(t^{\prime }+t\right)\right],$$

respectively, where $k_{B}$ is the Boltzmann constant and T is the absolute temperature. It is observed in [71] that when colloidal beads diffuse along linear phospholipid bilayer tubes or through entangled F-actinnetworks, the motion of beads show Brownian yet non-Gaussian dynamics. Later, the diffusing diffusivity microscopic model describing this kind of phenomena is built as [72]

(5) $$\begin{array}{c} \frac{dx(t)}{dt}=\sqrt{2D(t)}\xi \left(t\right), \end{array}$$

(6) $$\begin{array}{c} D\left(t\right)=y^{2}\left(t\right), \end{array}$$

(7) $$\begin{array}{c} \frac{dy(t)}{dt}=-y\left(t\right)+\eta \left(t\right), \end{array}$$

where, x(t) and y(t) (Ornstein-Uhlenbeck process) are stochastic processes driven by independent Gaussian white noises ξ(t) and η(t), respectively, and $D\left(t\right)=y^{2}\left(t\right)$ provides the diffusion coefficient for x(t). The reasons for choosing the square of the Ornstein-Uhlenbeck process as the diffusing diffusivity of x(t) are threefold. Firstly, the non-negativity of this choice avoids the need to add a reflecting boundary condition when D(t)=0, making the analysis easier to handle. Secondly, it ensures that the dynamics of the diffusivity is stationary under the given correlation time. Moreover, it also guarantees that the PDF of D(t) has exponential tails, resulting in a Laplace-like distribution for x(t) at short times. Over long times, the particle’s motion with an effective diffusion coefficient ${\langle D\rangle }_{st}=\lim_{t\to \infty }\langle y^{2}\left(t\right)\rangle$ exhibits a crossover to a normal distribution. The Brownian non-Gaussian diffusion induced by polymerization is discussed in [[9], [68]], being modelled by Eq. 5 with the diffusing diffusivity D(N(t)), where N(t) is the birth-death process satisfying

(8) $$\begin{array}{cc} & P\left(N(t+\tau )-N(t)=k|N(t)=n\right)\\ = & \left\{ \begin{array}{cc} \alpha (n)\tau +o(\tau ),\; & k=1,\\ \beta (n)\tau +o(\tau ), & k=-1,\\ 1-(\alpha (n)+\beta (n))\tau +o(\tau ), & k=0,\\ o(\tau ), & \text{otherwise}, \end{array}\right. \end{array}$$

with α(n),β(n)≥0 for n∈N, and β(0)=0.

#### 2.1.3 Miscellaneous processes

In probability theory, the technique of time-changing for Brownian motion is one of the important methods to build a new stochastic process. First, one needs to define a time change process, e.g., α-stable Lévy process $S_{\alpha }\left(t\right)$ with α∈(0,1), and its inverse $E_{\alpha }\left(t\right)$. Then, $B(S_{\alpha }\left(t\right))$ and $B(E_{\alpha }\left(t\right))$, which are the time change to Brownian motion B(t), can be respectively used to describe superdiffusion, being the 2α-stable Lévy process, and subdiffusion. In fact, one can also build the stochastic process $B(S_{\alpha }\left(E_{\beta }\left(t\right)\right))$ (β∈(0,1)) to characterize the competition between superdiffusion and subdiffusion.

One can build multiple-state alternating stochastic process, for example, a two-state alternating stochastic process [21], which alternates between Lévy walk and Brownian motion, i.e., the sojourn times in the Lévy walk and Brownian motion stages follow power-law distributions with exponents $\alpha_{+}$ and $\alpha_{-}$ $\left(0<\alpha_{\pm }<2\right)$, respectively. This kind of processes generally have several different scales and show strong anomalous diffusion phenomena [21].

Resetting process is needed in some practical applications, e.g., search processes [[73], [74], [75], [76]], stochastic algorithms [[77], [78]], catastrophic phenomena in population dynamics [[79], [80], [81]], etc. A concrete example is given as follows [25]: the position x(t) of the particle with an initial position of $x\left(0\right)=x_{0}$ at time t is updated by the stochastic rule

(9) $$x\left(t+dt\right)=\left\{ \begin{array}{cc} X_{r}, & \text{with}\;\text{probability}\;rdt,\\ x(t)+dB(t), & \text{with}\;\text{probability}\;(1-rdt), \end{array}\right.$$

where $X_{r}$ is a fixed position, r is the resetting rate, and B(t) is Brownian motion. This stochastic process describes a Brownian motion particle with Poissonian resetting, which, after a time interval dt, is reset to position $X_{r}$ with a probability of rdt, and undergoes Brownian motion with a probability of (1−rdt).

The motion in non-static media is an important research field. In biology, during the development of vertebrate embryos, cell migrations occur on an underlying tissue domain in response to some factor, such as nutrient. Over the time scale of days in which this cell migration occurs, the underlying tissue is itself growing. In this case, the media is an expanding one with scale factor a(t). Following the discussions from (1)-(3) to (4) in [82], the original Langevin equation is modified as

(10) $$\frac{dx(t)}{dt}=\frac{da(t)}{dt}\frac{x(t)}{a(t)}+\xi \left(t\right).$$

### 2.2 Macroscopic equations

This subsection presents the governing equations of the probability distributions of some typical statistical observations for the above built microscopic models.

#### 2.2.1 Fokker-Planck equations

Here we focus on the probability distribution of the position of the particles [[40], [47]]. The most well-known case is Brownian motion B(t), its Fokker-Planck equation behaves as

(11) $$\left\{ \begin{array}{c} \frac{\partial W(x,t)}{\partial t}=\Delta W\left(x,t\right),\\ W(x,t=0)=\delta (x). \end{array}\right.$$

If the particle performs subdiffusion, microscopically modelled by $B(E_{\alpha }\left(t\right))$ or the corresponding CTRW or Langevin picture, the governing equation of the probability distribution of its position is described as [47]

(12) $$\left\{ \begin{array}{c} \frac{\partial W(x,t)}{\partial t}={}_{0}D_{t}^{1-\alpha }\Delta W\left(x,t\right),\\ W(x,t=0)=\delta (x), \end{array}\right.$$

where ${}_{0}D_{t}^{1-\alpha }$ is the Riemann-Liouville operator defined as

$${}_{0}D_{t}^{1-\alpha }W\left(x,t\right)=\frac{1}{\Gamma \left(\alpha \right)}\frac{\partial }{\partial t}\int_{0}^{t}\frac{W\left(x,t^{\prime }\right)}{{\left(t-t^{\prime }\right)}^{1-\alpha }}dt^{\prime }.$$

Similarly, the superdiffusion of the particle can be microscopically modelled by $B(S_{\beta }\left(t\right))$, or through the corresponding CTRW or Langevin picture. Its governing equation of probability distribution of the position is given as [[83], [84]]

(13) $$\left\{ \begin{array}{c} \frac{\partial W(x,t)}{\partial t}=-{\left(-\Delta \right)}^{\beta }W\left(x,t\right),\\ W(x,t=0)=\delta (x), \end{array}\right.$$

where $F\left\{-{\left(-\Delta \right)}^{\beta }W\left(x,t\right)\right\}\left(k\right)=-{\left|k\right|}^{2\beta }F\left\{W\left(x,t\right)\right\}\left(k\right)$.

More general case including possible normal diffusion, subdiffusion, and superdiffusion can be microscopically described by the competition model $B(S_{\beta }\left(E_{\alpha }\left(t\right)\right))$. The corresponding macroscopic equation behaves as [[85], [86]]

(14) $$\left\{ \begin{array}{c} \frac{\partial W(x,t)}{\partial t}={}_{0}D_{t}^{1-\alpha }\left(-{\left(-\Delta \right)}^{\beta }\right)W\left(x,t\right),\\ W(x,t=0)=\delta (x). \end{array}\right.$$

There are also more generalized models of the space-time fractional diffusion equations by introducing general memory kernel [[87], [88], [89]]. The forward and backward Fokker-Planck equations for the polymer diffusion with the microscopic model Eq. 5 and Eq. 8 are respectively described as [[34], [68]]

(15) $$\left\{ \begin{array}{c} \frac{\partial W(n,x,t)}{\partial t}=L_{n}W\left(n,x,t\right)+D\left(n\right)\nabla_{x}^{2}W\left(n,x,t\right),\\ W(n,x,t=0)=g(n)\delta (x), \end{array}\right.$$

and

(16) $$\left\{ \begin{array}{c} \frac{\partial W_{n_{0}}\left(x,t\right)}{\partial t}=F_{n_{0}}W_{n_{0}}\left(x,t\right)+D\left(n_{0}\right)\nabla_{x}^{2}W_{n_{0}}\left(x,t\right),\\ W_{n_{0}}\left(x,t=0\right)=\delta \left(x\right), \end{array}\right.$$

where $L_{n}$ and $F_{n_{0}}$ are given difference operators defined as

$$L_{n}W\left(n,x,t\right)=\left\{ \begin{array}{cc} \mu (n+1)W(n+1,x,t)-\mu (n)W(n,x,t) & \\ +\lambda (n-1)W(n-1,x,t)-\lambda (n)W(n,x,t), & n>0,\\ \mu (1)W(1,x,t)-\lambda (0)W(0,x,t), & n=0, \end{array}\right.$$

and

$$F_{n_{0}}W_{n_{0}}\left(x,t\right)=\left\{ \begin{array}{cc} \lambda \left(n_{0}\right)\left(W_{n_{0}+1}\left(x,t\right)-W_{n_{0}}\left(x,t\right)\right)\\ +\mu \left(n_{0}\right)\left(W_{n_{0}-1}\left(x,t\right)-W_{n_{0}}\left(x,t\right)\right), & n_{0}>0,\\ \lambda \left(0\right)\left(W_{1}\left(x,t\right)-W_{0}\left(x,t\right)\right), & n_{0}=0. \end{array}\right.$$

#### 2.2.2 Feynman-Kac equations

We turn to the statistical observable A, which is the functional of the trajectory of a particle x(t), defined as

$$A=\int_{0}^{t}U\left(x\left(t^{\prime }\right)\right)dt^{\prime },$$

with U(x) being a given function. Two types of Feynman-Kac equations have been derived, respectively governing the joint distribution of A and x(t) (called forward Feynman-Kac equation) and the distribution of A with the process’s starting point $x\left(0\right)=x_{0}$ as the parameter (called backward Feynman-Kac equation).

First, we present the backward Feynman-Kac equation for the microscopic model with external potential and multiplicative noise. The model behaves as

(17) $$\frac{dx(t)}{dt}=-\nabla V\left(x\left(t\right)\right)+g\left(x\left(t\right)\right)\xi \left(t\right).$$

While ξ(t) can be white noise and β-stable Lévy noise (g(x)≡1), the backward Feynman-Kac equations of Eq. 17 are respectively given as [52]

(18) $$\left\{ \begin{array}{c} \begin{array}{cc} \frac{\partial G_{x_{0}}\left(p,t\right)}{\partial t} & =g^{2}\left(x_{0}\right)\Delta_{x_{0}}G_{x_{0}}\left(p,t\right)-\nabla V\left(x_{0}\right)\cdot \nabla_{x_{0}}G_{x_{0}}\left(p,t\right)\\ & \;-\;ipU\left(x_{0}\right)G_{x_{0}}\left(p,t\right), \end{array}\\ G_{x_{0}}\left(p,t=0\right)=1, \end{array}\right.$$

and

(19) $$\left\{ \begin{array}{c} \begin{array}{cc} \frac{\partial G_{x_{0}}\left(p,t\right)}{\partial t} & =-{\left(-\Delta_{x_{0}}\right)}^{\beta /2}G_{x_{0}}\left(p,t\right)-\nabla V\left(x_{0}\right)\cdot \nabla_{x_{0}}G_{x_{0}}\left(p,t\right)\\ & \;-\;ipU\left(x_{0}\right)G_{x_{0}}\left(p,t\right). \end{array}\\ G_{x_{0}}\left(p,t=0\right)=1. \end{array}\right.$$

In the following, we provide the forward and the backward Feynman-Kac equations for the microscopic models [[86], [90]]: $B(E_{\alpha }\left(t\right))$, $B(S_{\beta }\left(t\right))$, $B(S_{\beta }\left(E_{\alpha }\left(t\right)\right))$.

The forward Feynman-Kac equations:

(20) $$\left\{ \begin{array}{c} \frac{\partial G(x,p,t)}{\partial t}=\Delta D_{t}^{1-\alpha }G\left(x,p,t\right)-ipU\left(x\right)G\left(x,p,t\right),\\ G(x,p,t=0)=\delta (x), \end{array}\right.$$

(21) $$\left\{ \begin{array}{c} \frac{\partial G(x,p,t)}{\partial t}=-{\left(-\Delta \right)}^{\beta }G\left(x,p,t\right)-ipU\left(x\right)G\left(x,p,t\right),\\ G(x,p,t=0)=\delta (x), \end{array}\right.$$

and

(22) $$\left\{ \begin{array}{c} \frac{\partial G(x,p,t)}{\partial t}=-{\left(-\Delta \right)}^{\beta }D_{t}^{1-\alpha }G\left(x,p,t\right)-ipU\left(x\right)G\left(x,p,t\right),\\ G(x,p,t=0)=\delta (x), \end{array}\right.$$

which will reduce to the corresponding Fokker-Planck equations when taking p=0.

The backward Feynman-Kac equations:

(23) $$\left\{ \begin{array}{c} \frac{\partial G_{x_{0}}\left(p,t\right)}{\partial t}=D_{t}^{1-\alpha }\Delta_{x_{0}}G_{x_{0}}\left(p,t\right)-ipU\left(x_{0}\right)G_{x_{0}}\left(p,t\right),\\ G_{x_{0}}\left(p,t=0\right)=1, \end{array}\right.$$

(24) $$\left\{ \begin{array}{c} \frac{\partial G_{x_{0}}\left(p,t\right)}{\partial t}=-{\left(-\Delta_{x_{0}}\right)}^{\beta }G_{x_{0}}\left(p,t\right)-ipU\left(x_{0}\right)G_{x_{0}}\left(p,t\right),\\ G_{x_{0}}\left(p,t=0\right)=1, \end{array}\right.$$

and

(25) $$\left\{ \begin{array}{c} \frac{\partial G_{x_{0}}\left(p,t\right)}{\partial t}=D_{t}^{1-\alpha }\left(-{\left(-\Delta_{x_{0}}\right)}^{\beta }\right)G_{x_{0}}\left(p,t\right)-ipU\left(x_{0}\right)G_{x_{0}}\left(p,t\right),\\ G_{x_{0}}\left(p,t=0\right)=1. \end{array}\right.$$

The forward and backward Feynman-Kac equations for the polymer diffusion with the microscopic model Eq. 5 and Eq. 8 are respectively described as [[34], [68]]

(26) $$\left\{ \begin{array}{c} \begin{array}{cc} \frac{\partial G(n,x,p,t)}{\partial t} & =L_{n}G\left(n,x,p,t\right)+D\left(n\right)\nabla_{x}^{2}G\left(n,x,p,t\right)\\ & \;-\;ipU(x)G(n,x,p,t), \end{array}\\ G(n,x,p,t=0)=g(n)\delta (x). \end{array}\right.$$

and

(27) $$\left\{ \begin{array}{c} \begin{array}{cc} \frac{\partial G_{n_{0},x_{0}}\left(p,t\right)}{\partial t} & =F_{n_{0}}G_{n_{0},x_{0}}\left(p,t\right)+D\left(n_{0}\right)\nabla_{x_{0}}^{2}G_{n_{0},x_{0}}\left(p,t\right)\\ & \;-\;ipU\left(x_{0}\right)G_{n_{0},x_{0}}\left(p,t\right), \end{array}\\ G_{n_{0},x_{0}}\left(p,t=0\right)=1. \end{array}\right.$$

#### 2.2.3 Miscellaneous equations

The concept of first passage time, which is the moment when a random variable first attains a specific value, has many applications, e.g., a consideration for investors deciding when to buy or sell stocks during price fluctuations. Escape probability and maximum displacement are closely related concepts to the first passage time and share similar application scenarios. Consider the following stochastic process [53]

(28) $$\left\{ \begin{array}{c} \frac{dx(s)}{ds}=-\nabla V\left(x\left(s\right)\right)+g\left(x\left(s\right)\right)\xi_{2}\left(s\right),\\ \frac{dt(s)}{ds}=\theta \left(s\right), \end{array}\right.$$

where θ(s) and $\xi_{2}\left(s\right)$ are independent of each other, t(s) is a tempered α-stable Lévy process (θ(s) is the corresponding noise), with the characteristic function given by $\langle e^{-ut(s)}\rangle =e^{-s({\left(u+\lambda \right)}^{\alpha }-\lambda^{\alpha })}$, which results in a α-stable Lévy process when the control factor λ(≥0) approaches 0, and $\xi_{2}\left(s\right)$ is a β-stable Lévy noise.

The mean first passage time u(x) of the process defined in Eq. 28 from the boundary of Ω starting at the point x∈Ω satisfies [53]

(29) $$\begin{array}{c} \left\{ \begin{array}{cc} -\nabla V\left(x\right)\cdot \nabla u\left(x\right)-{\left(-\Delta \right)}^{\beta /2}u\left(x\right)=-\alpha \lambda^{\alpha -1}, & x\in \Omega ,\\ u\left(x\right)=0, & x\in \Omega^{c}. \end{array}\right. \end{array}$$

Then, we calculate the probability $p_{\Gamma }\left(x\right)$ of the jump process defined in Eq. 28, starting from the point x∈Ω, and firstly entering the domain Γ. The $p_{\Gamma }\left(x\right)$ is called escape probability solving

(30) $$\left\{ \begin{array}{c} -\nabla V\left(x\right)\cdot \nabla p_{\Gamma }\left(x\right)-{\left(-\Delta \right)}^{\beta /2}p_{\Gamma }\left(x\right)=0,x\in \Omega ,\\ p_{\Gamma }\left(x\right){{|}_{x\in \Gamma }=1,p_{\Gamma }\left(x\right)|}_{x\in \Omega^{c}\setminus \Gamma }=0. \end{array}\right.$$

The microscopic model Eq. 3 can be rephrased as

(31) $$\left\{ \begin{array}{c} \frac{dv(t)}{dt}=-\frac{\nabla V(x(t))}{m}-\gamma v\left(t\right)+\frac{\xi_{1}\left(t\right)}{m},\\ \frac{dx(t)}{dt}=v\left(t\right). \end{array}\right.$$

The joint distribution f(x,v,t) of x(t) and v(t) solve the equation [91]

(32) $$\frac{\partial f(x,v,t)}{\partial t}=\left[-v\frac{\partial }{\partial x}+\frac{\partial }{\partial v}\left(\gamma v+\frac{\nabla V(x)}{m}\right)+\frac{1}{m^{2}}\frac{\partial^{2}}{\partial v^{2}}\right]f\left(x,v,t\right),$$

which is called Klein-Kramers equation. While for the time changed microscopic model $v(E_{\alpha }\left(t\right))$ and $dx\left(t\right)=dv(E_{\alpha }\left(t\right))$, where v(t) is defined as the first equation of Eq. 31, its Klein-Kramers equation is

(33) $$\begin{array}{c} \frac{\partial f(x,v,t)}{\partial t}+v\frac{\partial }{\partial x}f\left(x,v,t\right)\\ \;={}_{0}D_{t}^{1-\alpha }\left[\frac{\partial }{\partial v}\left(\gamma v+\frac{\nabla V(x)}{m}\right)+\frac{1}{m^{2}}\frac{\partial^{2}}{\partial v^{2}}\right]f\left(x,v,t\right). \end{array}$$

## 3 Analysis and algorithm

In this section, we shall analyze the well-posedness of above derived equations in a general form and the regularity of their solutions if possible. Then we propose algorithms, classical numerical algorithms as well as deep learning algorithms, to solve these equations. The analysis of proposed algorithms is performed as well.

### 3.1 Macroscopic equation for the time-changed strong Markov process

All the above discussions start from Brownian motion. Here we discuss a more general form, starting from the strong Markov process X(t). Define the time-changed process Y(t)=X(E(t)), where E(t) is also extended to the inverse of more general subordinator with the characteristic function $e^{-t\phi (z)}$. It can be noted that when $\phi \left(z\right)=z^{\alpha }$, the subordinator is exactly the above mentioned α-stable Lévy process. The stochastic representation

(34) $$u\left(x,t\right)=E^{x}\left[e^{-\int_{0}^{t}\kappa \left(Y\left(s\right)\right)ds}f\left(Y\left(t\right)\right)\right]$$

is the unique mild solution to the equation [41]

(35) $$\left\{ \begin{array}{c} \partial_{t}^{w,\kappa (x)}u\left(x,t\right)=Lu\left(x,t\right)-\kappa \left(x\right)I_{t}^{w,\kappa (x)}u\left(x,t\right),\\ u(x,0)=f(x), \end{array}\right.$$

where L is the infinitesimal generator of Y(t). Moreover, the mapping t→u(·,t) is continuous with $∥u\left(\cdot ,t\right)∥\leq C_{1}e^{C_{2}t},C_{1},C_{2}>0$, and $\hat{u}\left(x,z\right)\;=\int_{0}^{\infty }e^{-zt}u\left(x,t\right)dt$, i.e., Laplace transform of u(x,t), exists and satisfies

(36) $$L\hat{u}\left(x,z\right)=\phi \left(z+U\left(x\right)\right)\hat{u}\left(x,z\right)-f\left(x\right)\frac{\phi (z+U(x))}{z+U(x)}.$$

When f≡1, $u_{x_{0}}\left(p,t\right)=E^{x_{0}}\left[e^{-ip\int_{0}^{t}U\left(Y\left(s\right)\right)ds}\right]$ solves the backward Feynman-Kac equation

(37) $$\left\{ \begin{array}{c} \partial_{t}^{w,\kappa (x_{0})}u_{x_{0}}\left(p,t\right)=Lu_{x_{0}}\left(p,t\right)-ipU\left(x_{0}\right)I_{t}^{w,\kappa (x_{0})}u_{x_{0}}\left(p,t\right),\\ u_{x_{0}}\left(p,0\right)=1. \end{array}\right.$$

Applying above results, one can calculate the PDFs of statistical observables (e.g., first passage time, occupation time and path integrals) relating to Y(t). The key here is to get u(x,t) from Eq. 35 and Eq. 36. We take first passage time of non-Markov anomalous subdiffusion as an example and refer interested readers to [41] for other cases. Precisely, for b∈R, define

$$\tau_{b}=\inf\left\{t>0:Y\left(t\right)>b\right\},$$

as the considered first passage time. Note that for ρ>0, we have

$$P^{x}\left(\tau_{b}>t\right)=P^{x}\left(\underset{0\leq s\leq t}{\sup}Y\left(s\right)<b\right)=\lim_{\rho \to \infty }u_{\rho }\left(x,t\right),$$

where

$$u_{\rho }\left(x,t\right)=E^{x}\left[e^{-\rho \int_{0}^{t}1_{\left(b,\infty \right)}\left(Y_{s}\right)ds}\right],$$

is a type of Eq. 34 with $\kappa \left(x\right)=\rho 1_{\left(b,\infty \right)}\left(x\right)$ and f(x)≡1. By subtle techniques and Eq. 36 (see [41] for details), it is found that

$${\hat{u}}_{\rho }\left(x,z\right)=\frac{1}{z}\left(1-\frac{\rho \sqrt{\phi (z+\rho )}}{\left(z+\rho \right)\left(\sqrt{\phi \left(z\right)+\sqrt{\phi (z+\rho )}}\right)}e^{\left(x-b\right)\sqrt{\phi (z)}}\right).$$

By letting ρ→∞ and taking inverse Laplace transform, one shall get the probability distribution function of $\tau_{b}$. For many problems, one cannot solve Eq. 35 and Eq. 36 analytically. Hence, one may apply typical numerical methods or fast numerical methods; see [[92], [93], [94], [95]] and references therein for constructions of numerical methods and corresponding error estimates.

### 3.2 Space fractional diffusion equation

Consider the two-dimensional space fractional diffusion equation with variable coefficients [[54], [55]]

(38) $$\begin{array}{ccc} \frac{\partial u(x,t)}{\partial t} & = & \left(d_{1}\left(x\right){}_{x_{L}}D_{x}^{\alpha }+d_{2}\left(x\right){}_{x}D_{x_{R}}^{\alpha }\right)u\left(x,t\right)\\ & & +\left(e_{1}\left(x\right){}_{y_{L}}D_{y}^{\beta }+e_{2}\left(x\right){}_{y}D_{y_{R}}^{\beta }\right)u\left(x,t\right)+f\left(x,t\right), \end{array}$$

where x:=(x,y),0<t≤T, α,β∈(1,2) are fractional orders, variable coefficients $d_{1},d_{2},e_{1},e_{2}\geq 0$, and f are given functions. Here, zero boundary condition and initial condition

$$u\left(x,0\right)=u_{0}\left(x\right),\;x\in \Omega :=\left(x_{L},x_{R}\right)\times \left(y_{L},y_{R}\right),$$

are imposed. Recall that ${}_{x_{L}}D_{x}^{\alpha }$ and ${}_{x}D_{x_{R}}^{\alpha }$ are left and right Riemann-Liouville fractional derivatives [96] defined by

$${}_{x_{L}}D_{x}^{\alpha }u\left(x,t\right):=\frac{1}{\Gamma (2-\alpha )}\frac{\partial^{2}}{\partial x^{2}}\int_{x_{L}}^{x}{\left(x-\xi \right)}^{1-\alpha }u\left(\xi ,y,t\right)d\xi$$

and

$${}_{x}D_{x_{R}}^{\alpha }u\left(x,t\right):=\frac{1}{\Gamma (2-\alpha )}\frac{\partial^{2}}{\partial x^{2}}\int_{x}^{x_{R}}{\left(\xi -x\right)}^{1-\alpha }u\left(\xi ,y,t\right)d\xi ,$$

respectively. Note that ${}_{y_{L}}D_{y}^{\beta }$ and ${}_{y}D_{y_{R}}^{\beta }$ can be similarly defined. To obtain high order numerical methods for above problems, one of the most important things is to construct high order approximations to left and right Riemann-Liouville fractional derivatives. We have proposed weighted shifted Grünwald-Letnikov discretization (WSGD) [55] and weighted shifted Lubich discretization (WSLD) [54] that achieve second order accuracy and fourth order accuracy respectively. Both methods obtain high order accuracy by assembling low order difference operators with appropriate weights and shifts. Since Grünwald-Letnikov difference operator can be regarded as a special case of Lubich difference operator (i.e., fractional linear multistep method [92]), we may present them in a unified way.

The fractional linear multistep method, which is proposed by Lubich in 1986 [92], can handle Riemann-Liouville type fractional derivatives well with uniform mesh. This method may be characterized by its generating function

$$\delta^{\alpha }\left(\zeta \right)={\left(\sum_{i=1}^{L}\frac{1}{i}{\left(1-\zeta \right)}^{i}\right)}^{\alpha },$$

where L≤6 and α>0. Note that

• α=1, it reduces to classical L+1 point backward difference formula;
• L=1, it is the same as Grünwald-Letnikov difference operator.

It is shown in [92] that the fractional linear multistep method typically gives L-th order accuracy. However, for time dependent problems, a direct application of this method to a space fractional operator with α∈(1,2) often results in an unstable numerical scheme. This can be overcomed by shifting the underlying fractional multistep method. Unfortunately, it reduces to first order accuracy for all possible L after shifting. Inspired by linear multistep method, we find that a linear combination of different shifted first order difference operators leads to high order accuracy.

More specifically, the shifted Lubich’s difference operator is defined as

$$A_{h,p}^{\alpha ,L}u\left(x\right):=h^{-\alpha }\sum_{k=0}^{\infty }q_{k}^{\alpha ,L}u\left(x-\left(k-p\right)h\right),$$

where $q_{k}^{\alpha ,L}$ is the coefficient of Taylor expansion (|ζ|≤1)

$$\delta^{\alpha }\left(\zeta \right)=\sum_{k=0}^{\infty }q_{k}^{\alpha ,L}\zeta^{k}.$$

It is only of first order accuracy if p≠0 as least for L≤2. Assembling the operators with different shifts, high order approximations can be constructed.

WSGD method [55] (L=1): A linear combination of two different shifts p,q gives

$${}_{L}D_{h,p,q}^{\alpha ,1}u\left(x\right)=\frac{\alpha -2q}{2(p-q)}A_{h,p}^{\alpha ,1}u\left(x\right)+\frac{2p-\alpha }{2(p-q)}A_{h,q}^{\alpha ,1}u\left(x\right).$$

Under appropriate assumptions, it is proved (mainly using Fourier transform) that this approximation achieves second order accuracy for left RL fractional derivatives. Similar results can be derived for right RL fractional derivatives; see Remark 2.5 in [55]. Combining this with Crank-Nicolson method gives a high order numerical scheme for space diffusion equation. The numerical scheme is proved to be unconditionally stable when (p,q)=(1,0) and (p,q)=(1,−1) as the eigenvalues of the matrices corresponding to the discretized operators have negative real parts. Moreover,

$$∥u^{n}-U^{n}∥\leq C\left(\tau^{2}+h_{x}^{2}+h_{y}^{2}\right),$$

where ∥·∥ denotes discrete $L_{2}$ norm, and $\tau ,h_{x},h_{y}$ are stepsizes. As demonstrated in [55], a linear combination of more than two shifts can achieve at least third order accuracy. However, the resulting numerical scheme may not be unconditionally stable for some α which greatly limits its usage.

WSLD method [54] (L=2): A linear combination of four different shifts. It can also be obtained by the following way.

• second order: a linear combination of two first order approximations (p≠q)
  $${}_{L}D_{h,p,q}^{\alpha ,2}u\left(x\right)=\frac{q}{p-q}A_{h,p}^{\alpha ,2}u\left(x\right)+\frac{p}{p-q}A_{h,q}^{\alpha ,2}u\left(x\right).$$
• third order: a linear combination of two second order approximations (rs≠pq)
  $${}_{L}D_{h,p,q,r,s}^{\alpha ,2}u\left(x\right)=w_{1}{\;}_{L}D_{h,p,q}^{\alpha ,2}u\left(x\right)+w_{2}{\;}_{L}D_{h,r,s}^{\alpha ,2}u\left(x\right),$$
  where $w_{1}=\frac{3rs+2\alpha }{3(rs-pq)}$ and $w_{2}=\frac{3pq+2\alpha }{3(pq-rs)}$.
• fourth order: a linear combination of two third order approximations
  $${}_{L}D_{h,p,q,r,s,\bar{p},\bar{q},\bar{r},\bar{s}}^{\alpha ,2}u\left(x\right)=w_{3}{\;}_{L}D_{h,p,q,r,s}^{\alpha ,2}u\left(x\right)+w_{4}{\;}_{L}D_{h,\bar{p},\bar{q},\bar{r},\bar{s}}^{\alpha ,2}u\left(x\right),$$
  where $w_{3}$ and $w_{4}$ are constants (see Theorem 2.4 of [54]).

Similar to [55], a Crank-Nicolson method is used to derive fully discrete numerical scheme. Applying similar techniques as in WSGD method, for some reasonable shifts, the proposed numerical scheme is shown to be unconditionally stable. Moreover,

$$∥u^{n}-U^{n}∥\leq C\left(\tau^{2}+h_{x}^{4}+h_{y}^{4}\right).$$

Following the above constructions, there are two possible extensions to get higher order accuracy: i) starting from fractional multistep method when L≥3; ii) using advanced correction techniques to recover high order of shifted operator for L≥2 and then assembling them appropriately.

### 3.3 Tempered fractional Laplacian

From [49], it is found that the PDF of above mentioned Lévy flight and tempered Lévy flight formally satisfies (see (20)-(22) and (31)-(33) in [49])

(39) $$\frac{\partial u(x,t)}{\partial t}=-{\left(-\Delta +\lambda \right)}^{\beta /2}u\left(x,t\right),\;x\in \Omega \subset R^{d},$$

where

$$-{\left(-\Delta +\lambda \right)}^{\beta /2}u=c_{d,\beta ,\lambda }\lim_{\epsilon \to 0}\int_{R^{d}\setminus B_{\epsilon }\left(x\right)}\frac{u(x,t)-u(y,t)}{e^{\lambda |x-y|}{\left|x-y\right|}^{d+\beta }}dy$$

is tempered fractional Laplacian of u, with $c_{d,\beta ,\lambda }=\frac{\Gamma (d/2)}{2\pi^{d/2}\left|\Gamma \left(-\beta \right)\right|}$ for λ>0 and $c_{d,\beta ,0}=\frac{\beta \Gamma ((d+\beta )/2)}{2^{1-\beta }\pi^{d/2}\Gamma \left(1-\beta /2\right)}$ for λ=0. There are more discussions relating to tempered fractional Laplacian which is beyond the scope of this work; see [97] and references therein for details.

Note that Eq. 39 is a time dependent problem. Hence, one needs to specify initial and boundary conditions to make it well defined. The initial condition can be easily specified as the value of u(x,0) in domain Ω. However, specifying the boundary condition is not trivial as the above mentioned Lévy process and tempered Lévy process have discontinuous paths. As a consequence, the majority of trajectories of these stochastic processes cannot hit the boundary ∂Ω. Therefore, one must account for the information of u(x,t) on $R^{d}\setminus \Omega$. In [49], generalized Dirichlet type and Neumann type boundary conditions are discussed. Here, we focus on the former one. As illustrated in [49], the appropriate generalized Dirichlet type boundary conditions (refer to (40)-(42) and (49)-(51) in [49]) are

$${u\left(x,t\right)|}_{R^{d}\setminus \Omega }=g\left(x,t\right),$$

where for some constants C,M>0 and small ϵ>0, when |x|≥M, g(x,t) should satisfy

$$\frac{\left|g(x,t)\right|}{{\left|x\right|}^{\beta -\epsilon }}<C,$$

if λ=0 and

$$\frac{\left|g(x,t)\right|}{e^{\left(\lambda -\epsilon )|x\right|}}<C,$$

if λ>0 respectively. Under proper assumptions of g(x,t), the well-posedness of above time dependent equation is proved for λ=0 (see (71)-(75) in [49]) and it is claimed that the results for the case λ>0 can be similarly derived.

In some situations, for particles undergoing Lévy flights or tempered Lévy flights, the mean first passage time of particles and escape probability of particles are important. They are closely related to the corresponding steady state equation of the time dependent Eq. 39 which is given by

(40) $$\left\{ \begin{array}{cc} -{\left(-\Delta +\lambda \right)}^{\beta /2}u\left(x\right)=f\left(x\right), & x\in \Omega ,\\ u(x)=g(x), & x\in R^{d}\setminus \Omega . \end{array}\right.$$

If f(x)=−1,g(x)=0, the solution is the mean first passage time of particles. If f(x)=0 and

$$g\left(x\right)=\left\{ \begin{array}{cc} 1, & x\in H\subset R^{d}\setminus \Omega ,\\ 0, & x\in (R^{d}\setminus \Omega )\setminus H, \end{array}\right.$$

the solution represents the probability that particles land in H after first escaping Ω.

Now, we discuss the well-posedness of Eq. 40 and the regularity of the solutions of Eq. 40 when g(x)=0. For simplicity, let us first introduce some notations that is adopted in [[98], [99]]. For 0<s<1, define

$$H^{s}\left(\Omega \right)=\{v\in L^{2}{\left(\Omega \right):|v|}_{H^{s}\left(\Omega \right)}<\infty \},$$

where

$${\left|v\right|}_{H^{s}\left(\Omega \right)}={\left(\int \int_{\Omega \times \Omega }\frac{{\left(v\left(x\right)-v\left(y\right)\right)}^{2}}{{\left|x-y\right|}^{d+2s}}dxdy\right)}^{1/2},$$

and

$$H^{1+s}\left(\Omega \right)=\{v\in H^{1}\left(\Omega \right):|\partial_{x_{i}}v\left|\in H^{s}\left(\Omega \right),1\leq i\leq d\right\}.$$

$H^{s}\left(\Omega \right)$ and $H^{1+s}\left(\Omega \right)$ are equipped with norm ${∥v∥}_{H^{s}\left(\Omega \right)}={∥v∥}_{L^{2}\left(\Omega \right)}+{\left|v\right|}_{H^{s}\left(\Omega \right)}$ and ${∥v∥}_{H^{1+s}\left(\Omega \right)}={∥v∥}_{H^{1}\left(\Omega \right)}+\sum_{i=1}^{d}{\left|\partial_{x_{i}}v\right|}_{H^{s}\left(\Omega \right)}$ respectively. Further, let

$$H^{s}\left(\Omega \right)={\left\{v\right|}_{\Omega }:v\in H^{s}\left(R^{d}\right),v{|}_{R^{d}\setminus \Omega }=0\},$$

and $H^{-s}\left(\Omega \right)$ be the dual of $H^{s}\left(\Omega \right)$.

For λ=0, by standard techniques, it is shown in [[98], [99]] that for $f\in H^{-\beta /2}\left(\Omega \right)$, there exists a unique solution $u\in H^{\beta /2}\left(\Omega \right)$ of the weak formulation of Eq. 40. Moreover, if $f\in H^{r}\left(\Omega \right)$ for some r≥−β/2, we shall get that $u\in H^{\beta /2+\gamma }\left(\Omega \right)$ with γ=min{β/2+r,1/2−ϵ} for arbitrarily small ϵ>0 and

$${∥u∥}_{H^{\beta /2+\gamma }\left(\Omega \right)}\leq C{∥f∥}_{H^{r}\left(\Omega \right)},$$

holds for smooth domain Ω. If Ω is not smooth (e.g., Lipschitz domain), then $u\in H^{\beta /2+1/2-\epsilon }\left(\Omega \right)$ and it holds that

$${∥u∥}_{H^{\beta /2+1/2-\epsilon }\left(\Omega \right)}\leq C{∥f∥}_{*},$$

where ${∥\cdot ∥}_{*}$ is some norm (see Theorem 3.3 in [99]). By defining appropriate weighted Sobolev spaces, an improved regularity result can be obtained (see Theorem 3.5 in [99]).

For λ>0, we consider d=1 [56]. Suppose that $f\in H^{-\beta /2}\left(\Omega \right)$, the weak formulation of Eq. 40 reads: find $u\in H^{\beta /2}\left(\Omega \right)$ such that

$$B\left(u,v\right)=\langle f,v\rangle ,\;\forall v\in H^{\beta /2}\left(\Omega \right),$$

where the duality pair is defined by $\langle f,v\rangle :=\int_{\Omega }fvdx$ and the bilinear form is given by

$$B\left(u,v\right):=\frac{c_{1,\beta ,\gamma }}{2}\int_{R}\int_{R}\frac{\left(u(x)-u(y))(v(x)-v(y)\right)}{e^{\lambda |x-y|}{\left|x-y\right|}^{1+\beta }}dxdy.$$

Similar to [98], using the Cauchy-Schwarz inequality, it is straightforward to show that the above bilinear form is continuous. Due to the existence of the term $e^{\lambda |x-y|}$, the method in [98] cannot be used directly to prove the coercivity of B(u,v). Fortunately, for Ω=(a,b), we are able to prove that B(u,v) is coercive by introducing some new ideas (see Propositions 3.2 and 3.3, Theorem 3.4 in [56]). Hence, by the Lax-Milgram theorem, Eq. 40 admits a unique solution. Based on the above results, a Riesz basis Galerkin numerical method is proposed in [56]. Using B-splines $M_{1}\left(x\right)$ and $M_{2}\left(x\right)$ [100], i.e.,

$$M_{1}\left(x\right)=\left\{ \begin{array}{cc} 1, & x\in [0,1],\\ 0, & \text{otherwise}, \end{array}\right.\;\text{and}\;M_{2}\left(x\right)=\int_{0}^{1}M_{1}\left(x-t\right)dt,$$

the approximation space $V_{n}^{r}$ (r=1 or r=2) is constructed as below

$$V_{n}^{r}=\left\{\phi_{n,j}^{r}\left(x\right)=2^{n/2}M_{r}\left(2^{n}x-j\right),j=0,1,\cdots ,2^{n}-r\right\},$$

for some n satisfying $2^{n}\geq 2r$. We aim to find the numerical solution $u_{n}\in V_{n}^{r}$ such that

$$B\left(u_{n},v_{n}\right)=\langle f,v_{n}\rangle ,\;\forall v_{n}\in V_{n}^{r}.$$

For $u\in H^{\mu }\left(\Omega \right)\cap H^{\beta /2}\left(\Omega \right)$ (μ≥β/2), it is shown that the numerical solution has the following approximation property

$$∥u-u_{n}{∥}_{H^{\beta /2}\left(R\right)}\leq C2^{-n(\min\mu ,r-\beta /2)}{∥u∥}_{H^{\beta /2}\left(\Omega \right)}.$$

### 3.4 Tempered fractional Feynman-Kac equation

Analyzing functional distribution of tempered anomalous dynamics is one of the feasible approaches to characterize it. As illustrated in Section 2, such a functional distribution is typically governed by the tempered fractional Feynman-Kac equation [[51], [57]]. In this part, we aim to introduce an efficient time discretization method for solving the following equation

(41) $$D_{t}\left(x\right)u\left(x,t\right)-\left(\lambda^{\alpha }+\Delta \right)D_{t}^{1-\alpha }u\left(x,t\right)=-u_{0}\left(x\right)\left(\lambda^{\alpha }D_{t}^{1-\alpha }-\lambda \right)e^{ipU(x)t}$$

with initial condition $u\left(x,0\right)=u_{0}\left(x\right),x\in \Omega \subset R^{d}$, and zero boundary condition. Here, p is the parameter representing the characteristic function of the joint probability of (x(t),A) with $A=\int_{0}^{t}U\left(x\left(\tau \right)\right)d\tau$, $D_{t}\left(x\right)$ is the substantial derivative given by

$$D_{t}\left(x\right):=\lambda -ipU\left(x\right)+\frac{\partial }{\partial t},$$

and $D_{t}^{1-\alpha }\left(0<\alpha <1\right)$ is the Riemann-Liouville fractional substantial derivative given by

$$D_{t}^{1-\alpha }u=\frac{1}{\Gamma (\alpha )}D_{t}\left(x\right)\int_{0}^{t}\frac{e^{-(t-s)(\lambda -ipU(x))}}{{\left(t-s\right)}^{1-\alpha }}u\left(x,s\right)ds.$$

Let us start with the well-posedness of Eq. 41. The analysis is based on the integral representation of u, which is derived mainly using Laplace transform and inverse Laplace transform. In fact, taking Laplace transform of Eq. 41 gives

(42) $$\left(\eta \left(z\right)-\Delta \right)\beta {\left(z\right)}^{1-\alpha }\hat{u}\left(x,z\right)=u_{0}\left(x\right)\beta {\left(z\right)}^{1-\alpha }\frac{\eta (z)}{z-ipU(x)},$$

where $\hat{u}\left(x,z\right)$ is the Laplace transform of u(x,t), β(z), and η(z) are given by

$$\beta \left(z\right)=z+\lambda -ipU\left(x\right)\;\text{and}\;\eta \left(z\right)=\beta {\left(z\right)}^{\alpha }-\lambda^{\alpha },$$

respectively. After getting an explicit expression of $\hat{u}\left(x,z\right)$ and then taking inverse Laplace transform, one obtains

$$u\left(x,t\right)=\frac{1}{2\pi i}\int_{\Gamma_{\theta ,\kappa }}e^{zt}\beta {\left(z\right)}^{\alpha -1}{\left(\eta \left(z\right)-\Delta \right)}^{-1}\left(\beta {\left(z\right)}^{1-\alpha }\frac{u_{0}\left(x\right)\eta \left(z\right)}{z-ipU(x)}\right)dz,$$

where $\Gamma_{\theta ,\kappa }$ is a contour (see (2.25) in [57]). Under appropriate assumptions, it is proved that the above integral representation is the mild solution of tempered fractional Feynman-Kac Eq. 41 (see Proposition 3.1(3) in [57]). One can note that the analysis is one of the main results in [57] and the integral expression is very important in the analysis of the numerical method in what follows.

Now we present the time-stepping scheme that is derived from the discretization in frequency domain, i.e., discretization based on Eq. 42. To this aim, we first construct an approximation of $D_{t}^{1-\alpha }u\left(x,t_{n}\right)$ in spatial domain. Then the final discretization is motivated by relating a transformation of this approximation to the Laplace transform of $D_{t}^{1-\alpha }u\left(x,t\right)$. More specifically, we have

• Approximation of $D_{t}^{1-\alpha }u\left(x,t_{n}\right)$. By straightforward calculations, it is observed that
  $$D_{t}^{1-\alpha }u\left(x,t\right)=e^{-t(\lambda -ipU(x))}{\left(\frac{\partial }{\partial t}\right)}^{1-\alpha }\left(e^{t(\lambda -ipU(x))u(x,t)}\right),$$
  where ${\left(\frac{\partial }{\partial t}\right)}^{1-\alpha }u$ is the standard Riemann-Liouville fractional derivative defined by
  $${\left(\frac{\partial }{\partial t}\right)}^{1-\alpha }u\left(x,t\right)=\frac{1}{\Gamma (\alpha )}\frac{\partial }{\partial t}\int_{0}^{t}{\left(t-s\right)}^{\alpha -1}u\left(x,s\right)ds.$$
  Approximating ${\left(\frac{\partial }{\partial t}\right)}^{1-\alpha }u$ by backward Euler convolution quadrature
  $${\bar{\partial }}_{\tau }^{1-\alpha }u_{n}=\frac{1}{\tau^{1-\alpha }}\sum_{j=1}^{n}b_{n-j}^{\left(1-\alpha \right)}u_{j},$$
  where τ is the time step size and $b_{n-j}^{\left(1-\alpha \right)}$ is the coefficient (see (2.5) in [57]), one can obtain an approximation of $D_{t}^{1-\alpha }u\left(x,t_{n}\right)$, that is,
  $${\bar{D}}_{\tau }^{1-\alpha }u_{n}\left(x\right):=e^{-t_{n}\left(\lambda -ipU\left(x\right)\right)}{\bar{\partial }}_{\tau }^{1-\alpha }\left(e^{t_{n}\left(\lambda -ipU\left(x\right)\right)u\left(x,t\right)}\right).$$
• Transformation of ${\bar{D}}_{\tau }^{1-\alpha }u_{n}\left(x\right)$. It is found that
  $$\sum_{n=1}^{\infty }{\bar{D}}_{\tau }^{1-\alpha }u_{n}\left(x\right)\zeta^{n}={\left(\frac{1-e^{-\tau (\lambda -ipU(x))}\zeta }{\tau }\right)}^{1-\alpha }\sum_{n=1}^{\infty }u_{n}\left(x\right)\zeta^{n}.$$
  Noticing that the Laplace transform of $D_{t}^{1-\alpha }u\left(x,t\right)$ is given by
  $$\begin{array}{cc} \beta {\left(z\right)}^{1-\alpha }\hat{u}\left(x,z\right) & =\int_{0}^{\infty }D_{t}^{1-\alpha }u\left(x,t\right)e^{-tz}dt\\ & \approx \tau \sum_{n=1}^{\infty }{\bar{D}}_{\tau }^{1-\alpha }u_{n}\left(x\right)e^{-t_{n}z}. \end{array}$$
  Taking $\zeta =e^{-\tau z}$ and comparing above results motivate us to consider the following approximations
  $$\beta \left(z\right)\approx \frac{1-e^{-\tau (z+\lambda -ipU(x))}}{\tau },\;\hat{u}\left(x,z\right)\approx \tau \sum_{n=1}^{\infty }u_{n}\left(x\right)e^{-t_{n}z}.$$
  We also apply above approximation of β(z) in η(z). For 1/(z−ipU(x)), we use $\tau e^{-\tau (z-ipU(x))}/\left(1-e^{-\tau (z-ipU(x))}\right)$ instead of $\tau /(1-e^{-\tau (z-ipU(x))})$ for analysis purpose.

Based on the above discussions, the resulting numerical scheme is obtained, that is,

$$\left({\bar{D}}_{\tau }^{\alpha }-\lambda^{\alpha }-\Delta \right){\bar{D}}_{\tau }^{1-\alpha }u_{n}\left(x\right)=\;u_{0}{\bar{D}}_{\tau }^{1-\alpha }\left({\bar{D}}_{\tau }^{\alpha }-\lambda^{\alpha }\right)e^{ipU\left(x\right)t_{n}},\;n\geq 1.$$

Applying Cauchy’s integral formula, we are able to get an integral expression of $u_{n}\left(x\right)$. With the help of explicit expressions of u(x,t) and $u_{n}\left(x\right)$, it is proved that (see Theorem 3.3 in [57])

$$∥u\left(\cdot ,t_{n}\right)-u_{n}{∥}_{M(\Omega )}\leq C{∥u_{0}∥}_{M(\Omega )}t_{n}^{-1}\tau ,\;n\geq 1,$$

where ${∥\cdot ∥}_{M(\Omega )}$ denotes the dual norm of $C(\bar{\Omega })$.

### 3.5 Equation driven by fractional Gaussion noise

Let us first briefly describe the equation considered in this part. Assume that Ω is a bounded domain, let B(t) be a standard Brownian motion with B(0)∈Ω and S(t) be a β-stable subordinator. Define a stochastic process X(t) as follows

$$X\left(t\right)=\left\{ \begin{array}{cc} B(S(t)), & S\left(t\right)\leq \tau_{\Omega },\\ \Theta , & S\left(t\right)\geq \tau_{\Omega }, \end{array}\right.$$

where $\tau_{\Omega }=\inf\left\{t>0:B\left(t\right)\notin \Omega \right\}$ is a stopping time of B(t) and Θ is a coffin state. From [101], it is seen that the infinitesimal generator of X(t) is the spectral fractional Laplacian operator ${\left(-\Delta \right)}^{\beta }$ (β∈(0,1)) that is defined by

$${\left(-\Delta \right)}^{\beta }u=\sum_{k=1}^{\infty }\lambda_{k}^{\beta }\left(u,\phi_{k}\right)\phi_{k},$$

where ${\left\{\lambda_{k},\phi_{k}\right\}}_{k=1}^{\infty }$ are eigenvalues and eigenfunctions (in $L^{2}\left(\Omega \right)$) pairs of −Δ with zero boundary condition. Then, the Fokker-Planck equation relating to X(t) time changed by the inverse α-stable subordinator is

$$\frac{\partial u}{\partial t}+{\left(\frac{\partial }{\partial t}\right)}^{1-\alpha }{\left(-\Delta \right)}^{\beta }u=0.$$

If there exists external fractional Gaussian noise and external source term depending on the density of particles, then we get the fractional diffusion equation driven by fractional Gaussian noise [58]

(43) $$\frac{\partial u}{\partial t}+{\left(\frac{\partial }{\partial t}\right)}^{1-\alpha }{\left(-\Delta \right)}^{s}u=f\left(u\right)+\dot{W_{Q}^{H}},\;x\in \Omega ,t\in \left(0,T\right]$$

with zero initial and boundary conditions. Here, f is a nonlinear term satisfying

$${∥f\left(u\right)∥}_{L^{2}\left(\Omega \right)}\leq {C(1+∥u∥}_{L^{2}\left(\Omega \right)}{),\;∥f\left(u\right)-f\left(v\right)∥}_{L^{2}\left(\Omega \right)}\leq C{∥u-v∥}_{L^{2}\left(\Omega \right)}.$$

$W_{Q}^{H}$ is the fractional Gaussian process defined by

$$W_{Q}^{H}=\sum_{k=1}^{\infty }\sqrt{\Lambda_{k}}\phi_{k}W_{k}^{H},$$

where ${\left\{W_{k}^{H}\right\}}_{k=1}^{\infty }$ are one dimensional fractional Brownian motions that are mutually independent, H∈(0,1) is the Hurst index, and Q is a nonnegative linear self-adjoint operator that has the same eigenfunctions with −Δ. The corresponding eigenvalues of Q are denoted by ${\left\{\Lambda_{k}\right\}}_{k=1}^{\infty }$.

Now we are ready to discuss the regularity of the mild solution of Eq. 43. First, an equivalent expression of the mild solution is given by taking Laplace transform and inverse Laplace transform, that is,

$$\begin{array}{cc} u & =\int_{0}^{t}R\left(t-r\right)f\left(u\left(r\right)\right)dr+\int_{0}^{t}R\left(t-r\right)dW_{Q}^{H}\left(r\right)\\ & =\int_{0}^{t}R\left(t-r\right)f\left(u\left(r\right)\right)dr+\sum_{k=1}^{\infty }\int_{0}^{t}\sqrt{\Lambda_{k}}E_{k}\left(t-r\right)\phi_{k}dW_{k}^{H}\left(r\right), \end{array}$$

where

$$R\left(t\right)=\frac{1}{2\pi i}\int_{\Gamma_{\theta ,\kappa }}e^{zt}z^{\alpha -1}{\left(z^{\alpha }+{\left(-\Delta \right)}^{s}\right)}^{-1}dz$$

and

$$E_{k}\left(t\right)=\frac{1}{2\pi i}\int_{\Gamma_{\theta ,\kappa }}e^{zt}z^{\alpha -1}{\left(z^{\alpha }+\lambda_{k}^{s}\right)}^{-1}dz$$

with $\Gamma_{\theta ,\kappa }$ being a contour as above.

Then applying estimates of R(t) and $E_{k}\left(t\right)$ (see (2.5) and (2.6) in [58]) and the regularization of noise (see Lemma 2.6 in [58])

$$E\left[{\left(\int_{0}^{T}g\left(T-r\right)dW_{k}^{H}\left(r\right)\right)}^{2}\right]\leq C{∥{\left(\frac{\partial }{\partial t}\right)}^{1/2-H}g∥}_{L^{2}\left(\left[0,T\right]\right)}^{2},$$

one can get the spatial regularity and temporal Hölder regularity of the mild solution u under appropriate assumptions, i.e.,

$$E\left[{∥{\left(-\Delta \right)}^{\sigma }u∥}_{L^{2}\left(\Omega \right)}^{2}\right]\leq C,\;E\left[{∥\frac{u(t)-u(t-\tau )}{\tau^{\gamma }}∥}_{L^{2}\left(\Omega \right)}^{2}\right]\leq C,$$

for some σ and γ (see Theorems 2.8 and 2.9 in [58]).

Next, we present the numerical method in [58]. The main idea is to apply spectral Galerkin method to discretize the fractional Laplacian and backward Euler convolution quadrature to discretize ${\left(\frac{\partial }{\partial t}\right)}^{1-\alpha }u$. The procedure is as follows:

1. Semidiscrete scheme. Using the above eigenfunctions, define a finite dimensional space $V_{N}$ as
  $$V_{N}=\text{span}\left\{\phi_{1},\cdots ,\phi_{N}\right\}\subset L^{2}\left(\Omega \right).$$
  Our objective is to find $u_{N}\left(t\right)\in V_{N}$ such that
  (44) $$\left\{ \begin{array}{c} \frac{\partial u_{N}}{\partial t}+{\left(\frac{\partial }{\partial t}\right)}^{1-\alpha }{\left(-\Delta \right)}_{N}^{s}u_{N}=P_{N}f\left(u_{N}\right)+P_{N}\dot{W_{Q}^{H}},\\ u_{N}\left(0\right)=0, \end{array}\right.$$
  where $P_{N}u=\sum_{i=1}^{N}\left(u,\phi_{i}\right)\phi_{i}$ is a projection of $L^{2}\left(\Omega \right)$ onto $V_{N}$ and ${\left(-\Delta \right)}_{N}^{s}:V_{N}\to V_{N}$ is defined by
  $$\left({\left(-\Delta \right)}_{N}^{s}u_{N},v_{N}\right)=\left({\left(-\Delta \right)}^{s}u_{N},v_{N}\right),\;\forall v_{N}\in V_{N}.$$
  Obviously, Eq. 44 has a similar form to Eq. 43. Hence, by similar techniques, one can obtain an explicit expression of $u_{N}\left(t\right)$ as well as the corresponding estimates. Further, one can get the spatial error
  $$E{\left[∥u-u_{N}{∥}_{L^{2}\left(\Omega \right)}^{2}\right]}^{1/2}\leq C{\left(N+1\right)}^{-2\sigma /d}.$$
2. Fully discrete scheme. The further discretization is based on Eq. 44. In fact, applying standard finite difference to $\frac{\partial u}{\partial t}$ and $\dot{W_{Q}^{H}}$ in Eq. 44, and backward Euler method to time fractional term ${\left(\frac{\partial }{\partial t}\right)}^{1-\alpha }$ in Eq. 44, one can get the fully discrete scheme of Eq. 43
  $$\partial_{\tau }u_{N}^{n}+{\bar{\partial }}_{\tau }^{1-\alpha }{\left(-\Delta \right)}_{N}^{s}u_{N}^{n}=P_{N}f\left(u_{N}^{n-1}\right)+P_{N}\partial_{\tau }W_{Q}^{H}\left(t_{n}\right),$$
  where $\partial_{\tau }u\left(t_{n}\right)=\frac{u\left(t_{n}\right)-u\left(t_{n-1}\right)}{\tau }$ and ${\bar{\partial }}_{\tau }^{1-\alpha }$ is the same as before.
  With the help of a proper transformation, one can also obtain an explicit expression of $u_{N}^{n}$ in a form similar to the one of $u_{N}\left(t\right)$. In this manner, for sufficiently small ϵ>0, one can derive the following temporal error
  $$E{\left[{∥u_{N}\left(t_{n}\right)-u_{N}^{n}∥}_{L^{2}\left(\Omega \right)}^{2}\right]}^{1/2}\leq C\tau^{H-\rho \alpha /s-\epsilon },$$
  where 0<ρ<min{sH/α,s} with 0<α<1.

Using the above derived spatial and temporal error, by triangle inequality, the numerical approximation error is given by

$$E{\left[{∥u\left(t_{n}\right)-u_{N}^{n}∥}_{L^{2}\left(\Omega \right)}^{2}\right]}^{1/2}\leq C\left({\left(N+1\right)}^{-2\sigma /d}+\tau^{H-\rho \alpha /s-\epsilon }\right).$$

### 3.6 Semilinear parabolic PDEs with infinite dimensional coupling

In [68], Fokker-Planck equations and Feynman-Kac equations that describe some statistical observables of polymer dynamics models are derived. These equations can be reformulated as semilinear parabolic PDEs with infinite dimensional coupling

(45) $$\left\{ \begin{array}{c} \frac{\partial u(n,x,t)}{\partial t}+T_{n}u\left(n,x,t\right)+T_{x}u\left(n,x,t\right)+f=0,\\ u(n,x,T)=g(n,x). \end{array}\right.$$

Here $u:N\times R^{d}\times \left[0,\infty \right)\to R$ is unknown, $T_{n}$ and $T_{x}$ are operators defined by

$$T_{n}u\left(n\right):=\left\{ \begin{array}{cc} \alpha (0)(u(1)-u(0)), & n=0,\\ \alpha (n)(u(n+1)-u(n))+\beta (n)(u(n-1)-u(n)), & n\geq 1, \end{array}\right.$$

where α(n),β(n) are given functions and

$$T_{x}:=\frac{1}{2}\text{Tr}\left(\left(\sigma \sigma^{T}\right)\left(n,x,t\right)Hess_{x}\right)+\mu \left(n,x,t\right)\cdot \nabla_{x}$$

with $\mu \in R^{d}$, $\sigma \in R^{d\times d}$ being vector-valued and matrix-valued functions, respectively, g and $f=f(t,n,x,u\left(n,x,t\right),\sigma^{T}\nabla_{x}u\left(n,x,t\right))$ being scalar-valued functions.

Because of the operator $T_{n}$, Eq. 45 becomes an infinite dimensional coupled lattice system. It is extremely difficult to solve it by traditional numerical methods if it is not impossible. Hence, we turn to deep neural network method. To be specific, we are going to extend standard deep BSDE method [[64], [65]] that works well for high dimensional nonlinear PDEs to infinite dimensional systems Eq. 45.

If there is no operator $T_{n}$, then standard deep BSDE method can be applied directly to Eq. 45. Roughly speaking, one needs three steps to construct a standard deep BSDE method [65], i.e.,

• Step 1: construct a stochastic process X(t),
  dX(t)=μdt+σdB(t),X(0)=x,
  the infinitesimal generator of which is exactly $T_{x}.$
• Step 2: derive a BSDE by applying Itô’s formula to u(X(t),t) first and then replacing u(X(t),t) and $\sigma \nabla_{x}u\left(X\left(t\right),t\right)$ by new notations Y(t) and Z(t). In this manner, one can get
  $$\begin{array}{cc} & dY\left(t\right)=-f\left(t,X\left(t\right),Y\left(t\right),Z\left(t\right)\right)dt+Z{\left(t\right)}^{T}dB\left(t\right),\\ & Y(T)=g(X(T)) \end{array}$$
  with $\left(Y\left(t\right),Z\left(t\right)\right)=(u\left(X\left(t\right),t\right),\sigma \nabla_{x}u\left(X\left(t\right),t\right))$ being the unique solution. This together with Step 1 gives so-called forward-backward stochastic differential equation (FBSDE)
  $$\left\{ \begin{array}{c} X\left(t\right)=X\left(0\right)+\int_{0}^{t}\mu ds+\int_{0}^{t}\sigma dB\left(s\right),\\ Y\left(t\right)=Y\left(T\right)+\int_{t}^{T}fds-\int_{t}^{T}Z{\left(s\right)}^{T}dB\left(s\right)\\ X(0)=x,\;Y(T)=g(X(T)), \end{array}\right.$$
  Hence, Eq. 45 without operator $T_{n}$ can be formulated as a constrained optimization problem below
  $$\underset{Y_{0},{\left\{Z_{t}\right\}}_{0\leq t\leq T}}{\inf}E\left[{\left|Y_{T}-g\left(X_{T}\right)\right|}^{2}\right],$$
  such that
  $$\left\{ \begin{array}{c} X\left(t\right)=X\left(0\right)+\int_{0}^{t}\mu ds+\int_{0}^{t}\sigma dB\left(s\right),\\ Y\left(t\right)=Y\left(0\right)-\int_{0}^{t}fds+\int_{0}^{t}Z{\left(s\right)}^{T}dB\left(s\right). \end{array}\right.$$
• Step 3: solve above optimization problem by discretizing constraints and approximating Y(0) and Z(t) via independent neural networks. Since Y(0)=u(X(0),t=0)=u(x,0), one can get an approximation of u(x,0).

The deep learning method in [68] follows a similar procedure as above and handles issues incurred by operator $T_{n}$. The first issue is to find an appropriate stochastic process whose infinitesimal generator is $T_{n}$. Unfortunately, it is not easy to find such a process. This may be overcomed partially by its microscopic description, i.e., the birth-death process N(t) that satisfies Eq. 8. Since N(t) is essentially a jump process, we are not able to eliminate $T_{n}$ totally as $T_{x}$. In fact, applying Itô’s formula to u(N(t),X(t),t), one can get the following BSDE (see Lemma 2.1 and Theorem 2.1 in [68])

(46) $$\begin{array}{cc} du(N(t),X(t),t)= & -fdt+{\left[\nabla_{x}u\right]}^{T}\sigma dB\left(t\right)+\\ & \int_{Z\setminus \{0\}}\delta u\left(t,n;N\left(t-\right)\right)\tilde{J}\left(dt,dn;N\left(t-\right)\right) \end{array}$$

with terminal condition u(N(T),X(T),T)=g(N(T),X(T)). Here δu(t,n;N(t−))=u(N(t−)+n,X(t),t)−u(N(t−),X(t),t) and $\tilde{J}\left(dt,dn;N\left(t-\right)\right)$ is a compensated counting random measure (see (2.4)-(2.6) in [68]). Obviously, one can approximate u(N(0),X(0),0) and ${\left[\nabla_{x}u\right]}^{T}\sigma$ in Eq. 46 by independent neural networks as in Step 3.

The remaining term in Eq. 46 is the second issue that we need to deal with. It can be noted that for the above birth-death process

$$\begin{array}{ccc} & & \int_{Z\setminus \{0\}}\delta u\left(t,n;N\left(t-\right)\right)\tilde{J}\left(dt,dn;N\left(t-\right)\right)\\ & = & [u(N(t),X(t),t)-u(N(t-),X(t),t)]dt\\ & & \;-\alpha (N(t-))[u(N(t-)+1,X(t),t)-u(N(t-),X(t),t)]dt\\ & & \;-\beta \left(N\left(t-\right)\right)[u\left(N\left(t-\right)-1,X_{t},t\right)-u\left(N\left(t-\right),X\left(t\right),t\right)]dt, \end{array}$$

where u(N(t−)±1,X(t),t) are unknown. One may also approximate them using neural networks as in Step 3. For simplicity, we devise a vector-valued neural network to approximate δu(t,±1;N(t−)) directly.

Till now, we have addressed two main issues encountered when applying deep BSDE method to Eq. 45. The full deep BSDE method for Eq. 45 can be derived easily following steps 1–3. We omit details here and refer interested readers to our work [68].

## 4 Applications in chemistry and biology

### 4.1 Modeling telomere shortening process

Aging is a complex biological process influenced by genes, environment, and lifestyle; and investigating its molecular and cellular changes can uncover potential mechanisms and intervention strategies. Telomeres are specific DNA sequences at the ends of linear chromosomes, and their shortening is associated with cellular aging, death, and cancer. However, some cells combat this process by expressing telomerase to repair and lengthen telomeres [[102], [103]]. Telomere shortening (TS) is primarily caused by incomplete replication of chromosomes, the action of exonucleases, and damage induced by oxidative stress; this application of above discussions will simulate the dynamic behavior of telomere length, derive macroscopic equations, and calculate the distribution of relevant statistical measures [104].

In the medical field, the measurement of telomere length serves as a crucial diagnostic tool [105]. To comprehend the complex processes of TS at the microscopic level, researchers have examined the underlying mechanisms of telomere length dynamics from a stochastic perspective.

According to [7], incomplete replication of chromosome ends leads to TS, with the shortened length $L_{1}$ following a normal distribution. The probability of TS due to exonuclease activity is measured/assumed to be 0.8, and the shortened length $L_{2}$ follows a Poisson distribution. During cellular replication, oxidative stress causes DNA damage to telomeres, leading to a measured/assumed probability of TS of 0.1, and the shortened length $L_{3}$ also follows a normal distribution. Then, there holds

(47) $$L=1\times \frac{1}{4}\times L_{1}+0.8\times \frac{1}{4}\times L_{2}+0.1\times \frac{1}{2}\times L_{3}\times N,$$

where L represents the shortened length of the telomere and N is the number of bases damaged in the DNA strand.

It is assumed that the waiting time adheres to a tempered power-law distribution, and that the TS jump length L changes independently at each step. Considering the low probability of TS due to oxidative stress damage, only the effects of incomplete replication at chromosome ends and exonuclease activity are taken into account. Therefore, $L=L_{1}+L_{2}$, and it is assumed that $L_{1}$ and $L_{2}$ are independent. Then, one can get the PDF of L as

(48) $$\begin{array}{cc} \varphi (L)= & \varphi_{1}\left(L_{1}\right)*\varphi_{2}\left(L_{2}\right)\\ ∼ & \sum_{L_{2}=0}^{\infty }\left[\frac{1}{\sqrt{2\pi }\sigma }\exp\left\{-\frac{{\left(L-\mu -L_{2}\right)}^{2}}{2\sigma^{2}}\right\}\times \exp\left\{-\eta \right\}\frac{\eta^{L_{2}}}{L_{2}!}\right], \end{array}$$

where “*” represents the convolution operation, and η is the intensity of the Poisson distribution, μ and σ are the mean and variance of the normal distribution, respectively.

Considering that the functional of length L is the conditional probability density G of $L\left(0\right)=L_{0}$, one can derive the backward Feynman-Kac equation [104]

(49) $$\begin{array}{cc} & \frac{\partial G_{L_{0}}\left(p,t\right)}{\partial t}\\ = & D_{t}^{1-\alpha ,\lambda }\left[\frac{\left(\eta +\mu \right)\left(1+B_{\alpha }\lambda^{\alpha }\right)}{B_{\alpha }}\frac{\partial }{\partial L_{0}}+\frac{\sigma^{2}}{2B_{\alpha }}\frac{\partial^{2}}{\partial L_{0}^{2}}+\lambda^{\alpha }\right]G_{L_{0}}\left(p,t\right)\\ & -\left[\lambda +pU(L_{0})\right]G_{L_{0}}\left(p,t\right)+\left(\lambda -\lambda^{\alpha }D_{t}^{1-\alpha ,\lambda }\right)e^{-pU(L_{0})t}. \end{array}$$

Since telomere length is not infinite at the onset of a cell’s life but starts with an initial length of $l_{0}$. When telomeres shorten to a certain degree, the stability of the genome within the cell is compromised, ultimately leading to cellular aging, death, or cancer. Specifically, when the length of the shortest telomere in the cell reaches the critical threshold $l_{c}$, the cell’s capacity for division becomes restricted and it begins to senesce. Therefore, the upper bound of the shortened length is $l_{0}-l_{c}$.

The occupation time is the total time for a telomere to shorten the length between $\left[0,l_{0}-l_{c}\right]$ in the observation time [0,t], which can be defined as [104]

(50) $$T^{+}=\int_{0}^{t}U\left(L\left(\tau \right)\right)\tau ,$$

where

(51) $$U\left(L\right)=\left\{ \begin{array}{cc} 1, & L\in [0,l_{0}-l_{c}],\\ 0, & L\notin [0,l_{0}-l_{c}]. \end{array}\right.$$

Exploring the total time of TS deepens our insight into cell aging mechanisms. TS is closely tied to the development of diseases like cancer, cardiovascular issues, and neurological conditions, offering key clues for prevention and treatment strategies. This application aids in anti-aging research and understanding disease progression. In [104], Figure 4 shows J(t) peaking before declining to zero, indicating the time most cell telomeres reach $l_{c}$. Due to the monotonically decreasing distribution of TS jump lengths, the occupancy time shares the same shape as the distribution of the first passage time.

### 4.2 Time-changed tempered fractional Langevin-Brownian motion

In certain real-world datasets, such as those in biology [[106], [107]], financial time series [108], ecology [109], and physics [110], a time-changed stochastic process is required. This process involves substituting the deterministic time variable with a positive, non-decreasing random process, which results in a blend of two independent random processes. One of these processes is referred to as the external process (or the original process), while the other is known as the internal process (or a subordinator).

Tempered fractional Langevin equation is driven by tempered fractional Gaussian noise γ(t) [44]. It is also a Gaussian process and can be written as

(52) $$\left\{ \begin{array}{c} \frac{dx(s)}{ds}=v\left(s\right),\\ \frac{dv(s)}{ds}=-\int_{0}^{s}K\left(s-\tau \right)v\left(\tau \right)d\tau +\rho \gamma \left(s\right),\\ \frac{dt(s)}{ds}=\eta \left(s\right), \end{array}\right.$$

where $\rho =\sqrt{2k_{B}T}$, the kernel

$$K\left(t\right)=2\langle \gamma \left(0\right)\gamma \left(t\right)\rangle =h^{-2}(C_{t+h}^{2}{\left|t+h\right|}^{2H}+C_{t-h}^{2}{\left|t-h\right|}^{2H}-2C_{t}^{2}{\left|t\right|}^{2H})$$

for a sufficient small h,

$$C_{t}^{2}=\frac{2\Gamma (2H)}{{\left(2\lambda |t|\right)}^{2H}}-\frac{2\Gamma \left(H+\frac{1}{2}\right)K_{H}\left(\lambda |t|\right)}{\sqrt{\pi }{\left(2\lambda |t|\right)}^{H}},$$

and $K_{H}\left(t\right)$ is the modified Bessel function of second kind. We assume the initial velocity satisfies the condition $v_{0}^{2}=k_{B}T$.

The PDF of the subordinated process X(t):=x(s(t)) can be written as

(53) $$p\left(x,t\right)=\int_{0}^{\infty }p_{0}\left(x,s\right)f\left(s,t\right)ds,$$

where $p_{0}\left(x,s\right)$ is the PDF of the original process x(s) and f(s,t) is the PDF of the inverse β-stable subordinator s(t). The moments of subordinated process X(t) could be obtained by the relation

(54) $$L_{t\to u}\langle X^{n}\left(t\right)\rangle =u^{\beta -1}L_{s\to u^{\beta }}\langle x^{n}\left(s\right)\rangle$$

in Laplace space. According to Eq. 54, with the time evolution the first and second moments of the subordinated process X(t):=x(s(t)) behave as

(55) $$\langle X\left(t\right)\rangle :\frac{\sqrt{k_{B}T}}{\beta \Gamma (\beta )}t^{\beta }$$

and

(56) $$\langle X^{2}\left(t\right)\rangle :\frac{k_{B}T}{\beta \Gamma (2\beta )}t^{2\beta }\to Ft^{(2-2H)\beta }\to \frac{\sqrt{k_{B}T}A}{\beta \Gamma (2\beta )}t^{2\beta },$$

where $E=\sqrt{k_{B}T}/\left[2D_{H}\Gamma^{2}\left(H+1/2\right)\Gamma \left(2H+1\right)\Gamma \left(\left(1-2H\right)\beta +1\right)\right]$ and $F=k_{B}T/\left[D_{H}\Gamma^{2}\left(H+1/2\right)\Gamma \left(2H+1\right)\Gamma \left(\left(2-2H\right)\beta +1\right)\right]$. The MSD of the time-changed tempered fractional Langevin equation evolves over time as [46]

(57) $$\begin{array}{cc} \langle {\left(\Delta X\left(t\right)\right)}^{2}\rangle : & \left(\frac{k_{B}T}{\beta \Gamma (2\beta )}-\frac{k_{B}T}{\beta^{2}\Gamma^{2}\left(\beta \right)}\right)t^{2\beta }\to Ft^{(2-2H)\beta }-E^{2}t^{2(1-2H)\beta }\\ & \to \left(\frac{\sqrt{k_{B}T}A}{\beta \Gamma (2\beta )}-\frac{A^{2}}{{\left(\beta \Gamma \left(\beta \right)\right)}^{2}}\right)t^{2\beta }. \end{array}$$

This implies that the time-changed tempered fractional Langevin process exhibits different characteristics at different time scales.

## 5 Future prospects

### 5.1 Biological macromolecules dynamics

The four most essential macromolecules in living organisms are nucleic acids, proteins, polysaccharides, and lipids. Materials like plastics, rubber, and fibers, which are types of polymer materials, have dramatically changed our daily life. These substances are created through the processes of polymerization and depolymerization from similar monomers. When studying the kinetic behavior of these materials, it’s crucial to take into account not only the polymer’s inherent movement characteristics but also the effects of chemical interactions during polymerization and depolymerization, as well as the influence of the surrounding environment. To delve deeper into the kinetic behavior of cell division and intact polymer proteins within living organisms, the following research initiatives are planned for future exploration.

#### 5.1.1 Kinetic modeling of microtubules

The dynamics and control of microtubules are vital for the proper functioning and division of all eukaryotic cells [[111], [112]]. As depicted in [111], microtubules extend and attach to the replicated DNA, forming a spindle and generating the pulling force that initiates cell division. Therefore, investigating the growth and regulation at the ends of microtubules can enhance our understanding of the mitotic behavior in eukaryotic cells.

#### 5.1.2 Protein synthesis, transport, and movement

Protein serves as a fundamental polymer in the human body, and its synthesis is a direct outcome of gene expression, which encompasses a series of processes such as transcription and translation. During the gene transcription process, RNA polymerase II moves along the DNA sequence, synthesizing mRNA. This movement can exhibit three distinct phases: transcriptional elongation, backtracking, and regressive recovery [113]. The translation process takes place in the ribosomes, hence requiring the transportation of the transcribed mRNA from the nucleus to the intended location following the completion of transcription [4]. The diffusion of synthesized proteins, like neurotransmitter receptors, on the surface of cell membranes can be influenced by crowded environments (as depicted in [5]). Therefore, it is both interesting and important to consider the modeling of the periodic behavior of proteins.

### 5.2 Multi-fluid modeling

#### 5.2.1 Modeling sediment transport by wind

The collective process of sand and dust being emitted, transported, and deposited by the wind is known as aeolian processes, named after the Greek god Aeolus, who was the keeper of the winds [[114], [115]]. Aeolian processes occur in areas where there is an ample supply of granular material and winds of sufficient force to move them through the atmosphere. On Earth, this phenomenon is most pronounced in deserts, on beaches, and in other areas with sparse vegetation, such as dried-up lake beds. The blowing of sand and dust in these regions plays a pivotal role in shaping the landscape through the formation of sand dunes and ripples, the erosion of rocks, and the creation and transport of soil particles. Furthermore, airborne dust particles can be carried for thousands of kilometers from their original source, impacting weather and climate, ecosystem productivity, the hydrological cycle, and various other components of the Earth’s system. Consequently, the study of the kinetic behavior of wind-blown sand is a significant area of research.

#### 5.2.2 Modeling fluid and solid interaction

The study of microclimates within plant canopies has long been a source of inspiration for scientists engaged in diverse research fields, including agronomy, ecology, and silviculture. It was nearly a century ago that the first measurements of wind speed within a forest stand were published in [116]. The behavior of wind in the canopy is an important component of the canopy microclimate, which largely determines the rate of exchange of heat, water vapor, and other associated gases and particles with the atmosphere. Consequently, the second topic of study is the interactions in the canopy and the wind field, which can be of great assistance in wind and sand control, seed dispersal [117], and in understanding inversions in agriculture.

#### 5.2.3 Modeling wind and fluid interaction, and the aroused enhanced diffusion

Ocean-atmosphere interactions exert a significant influence on the marine environment. For instance, hurricanes can impact upper ocean temperatures [[118], [119]], while interactions between ocean currents and winds affect surface carbon concentrations and air-sea carbon exchange in the Southern Ocean [120]. Global warming can be interrupted by the Pacific circulation [121], and the interactions between the ocean and wind can directly impact the dispersion of marine pollutants [122] and more. Therefore, general coupled ocean-atmosphere and pollutant dispersion modeling is an important and intriguing research topic.

### 5.3 General form of chemotaxis model

The myxobacteria are ubiquitous soil bacteria that aggregate under conditions of starvation and construct fruiting bodies as a means of survival. The mechanisms underlying their social gliding, aggregation, and fruiting body formation have remained poorly understood until recently. In [123], a stochastic cellular automaton model is presented with the objective of describing and providing an understanding of the mechanisms by which the bacteria manage to build higher-organized structures.

This model is affected by three factors which are, slime, diffusing chemoattractant, and inertia of motion. Therefore, an interesting topic is to derive the equations satisfied by the statistical observables of this chemotaxis model. By studying the equations, one can well understand the chemotaxis phenomena.

## Declaration of competing interest

The authors declare that they have no conflicts of interest in this work.
